# Equilibrium contrast cardiovascular magnetic resonance shows increased interstitial expansion in the systemic right ventricle of adults late after Mustard or Senning surgery for transposition of the great arteries

**DOI:** 10.1186/1532-429X-14-S1-O58

**Published:** 2012-02-01

**Authors:** Daniel Sado, Carla Plymen, Pier Lambiase, Aidan Bolger, Andrew M Taylor, Marina Hughes, James Moon

**Affiliations:** 1 Imaging Centre, The Heart Hospital, London, UK; 2 Cardiology, The Heart Hospital, London, UK; 3 Cardiovascular Magnetic Resonance, Great Ormond Street, London, UK; 4 East Midlands Congenital Heart Centre, Glenfield Hospital, Leicester, UK

## Summary

In this study, we have used equilibium contrast CMR (EQ-CMR) to assess the interstium volume of patients after atrial redirection surgery, finding higher amounts than in healthy volunteers and correlations with known clinical markers of disease.

## Background

After atrial redirection surgery (the Mustard and Senning operations) for transposition of the great arteries (TGA) the systemic right ventricle (RV) suffers from late systolic failure with resultant high morbidity and mortality. The mechanisms of late systemic RV failure are poorly characterised. We hypothesised that diffuse interstitial expansion representing diffuse fibrosis would be greater in the right ventricles of patients following Mustard or Senning surgery and that it would be associated with other markers of heart failure and disease severity in these patients.

## Methods

We used Equilibrium Contrast CMR to quantify interstitial expansion,Vd(m), in the septum and RV free wall of 14 consecutive adults presenting to a tertiary specialist clinic late after palliative surgery for TGA (8 Mustard, 6 Senning, 6 female, median age 33 (IQR: 7)). These were compared to an equal number of age and sex matched healthy volunteers from a denominator normal population of 86. Patients were also assessed with a standardised CMR protocol, NT-proBNP and cardiopulmonary exercise (CPEX) testing.

## Results

**Figure 1:**
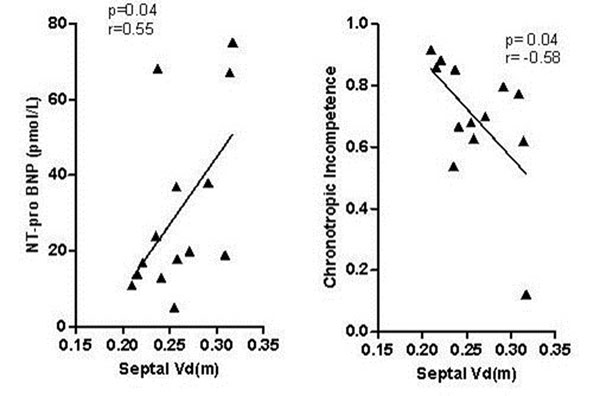
Significant relationship of septal Vd(m) with NT-proBNP (left panel) and chronotropic incompetence (right)

Mean septal Vd(m) was significantly higher in the patients than in the control population (0.254±0.036, vs 0.230±0.032; p= 0.03). NT-proBNP was positively related to septal Vd(m) (p=0.04; r=0.55, Figure 1). Chronotropic index (a measure of age corrected increase in heart rate on exercise) during CPEX testing was negatively related to Vd(m) (p=0.04; r=-0.58 Figure 1). No relationship was seen with other CMR or CPEX parameters.

RV free wall Vd(m) was difficult to measure (heavy trabeculation, sternal wires, blood pool in regions of interest) with high and poor inter-observer reproducibility such that this analysis was abandoned.

## Conclusions

In this small preliminary study, septal interstitial expansion is seen in adults late after atrial redirection surgery for TGA. It correlates well with NT-proBNP and chronotropic incompetence and may have a role in the development of RV systolic impairment. Measuring interstitial expansion in the RV free wall is difficult using this methodology.

## Funding

British Heart Foundation.

